# OP23 Key Challenges For The Appraisal Of Disease-Modifying Dementia Drugs

**DOI:** 10.1017/S0266462324000862

**Published:** 2025-01-07

**Authors:** Roshni Joshi, Dalia Dawoud, Fatima Salih, Tuba Saygin Avsar, Carole Longson, Nick Crabb

## Abstract

**Introduction:**

Disease-modifying dementia treatments (DMDTs) target amyloid beta or tau proteins and have the potential to change disease progression, representing a step change in the management and treatment of Alzheimer’s disease. Given the novel mechanism of action and impact on health care, the NICE Health Technology Assessment Innovation Laboratory (HTA Lab) sought to identify and contextualize the key issues for future appraisals.

**Methods:**

We reviewed published assessment reports of DMDTs from international HTA agencies and conducted a scoping review of published economic models of pharmacological treatments to understand the challenges associated with evaluating the cost-effectiveness of dementia treatments. The HTA Lab held an engagement workshop with 27 external stakeholders, including expert clinicians, implementation partners, health economists, and representatives from international agencies to discuss and confirm the key issues and considerations likely to emerge during an appraisal of DMDTs.

**Results:**

Key clinical and cost-effectiveness issues were identified and discussed. We concluded that consideration needs to be given to the diagnostic methods to identify the DMDT-eligible population in the UK, the validity of the surrogate outcomes used in the DMDT clinical trials, treatment effectiveness in different populations, and the incidence of DMDT-associated adverse events. Economic considerations include the type of economic model used in the appraisal, modeling the natural history of the disease, paucity of quality-of-life data in the treatment population, the inclusion of societal impact, treatment duration, stopping rules, and long-term effectiveness beyond the clinical trials.

**Conclusions:**

DMDTs could have the potential to transform Alzheimer’s disease care. With multiple treatments on the horizon, the appropriateness and acceptability of the new mechanism of action underpinning these treatments should be considered. We have identified areas of uncertainty that are likely to arise during an appraisal process, to facilitate the timely approval of these medicines and patient access.

